# Early and long-term outcomes of minimally invasive mitral valve surgery through right minithoracotomy: a 10-year experience in 1604 patients

**DOI:** 10.1186/s13019-015-0390-y

**Published:** 2015-12-07

**Authors:** Mattia Glauber, Antonio Miceli, Daniele Canarutto, Antonio Lio, Michele Murzi, Daniyar Gilmanov, Matteo Ferrarini, Pier A. Farneti, Eugenio L. Quaini, Marco Solinas

**Affiliations:** Cardiothoracic department, Fondazione Toscana G. Monasterio, Via Aurelia Sud, Massa, Italy

**Keywords:** Minimally invasive surgery, Mitral valve

## Abstract

**Background:**

To report early and long-term outcomes of patients undergoing minimally invasive mitral valve surgery (MIMVS) through right mini-thoracotomy (RT) over a 10-year period.

**Methods:**

From September 2003 to December 2013, a total of 1604 consecutive patients underwent MIMVS through RT.

**Results:**

The mean age was 63 ± 13 years, 770 (48 %) patients were female and 218 (13.6 %) had previous cardiac operations. The most predominant pathology was degenerative disease (70 %), followed by functional mitral valve regurgitation (12 %), rheumatic disease (9.4 %), endocarditis (5 %) and prosthetic dysfunction (3.2 %). Mitral valve repair was performed in 1137 (71 %) patients and 476 (29 %) had mitral valve replacement. Direct aortic cannulation was achieved in 1325 (83 %) patients. Among patients with degenerative disease candidate for repair (*n* = 958), rate of mitral valve repair was 95 %. Repair techniques included annuloplasty (95 %), leafleat resection (63 %), neochordae implantation (16 %) and sliding plasty (11 %). Concomitant procedures included tricuspid valve repair (14.6 %), atrial fibrillation ablation (9.5 %) and atrial septal defect closure (3.2 %). Overall in-hospital mortality was 1.1 %. Thirty-four patients (2.1 %) had conversion to sternotomy. Incidence of stroke was 2 %. Overall survival at 10 years was 88 ± 2 %. Freedom from reoperation at 10 years was 94 ± 2 % for repair and 80 ± 6 % for replacement. Freedom from recurrent mitral regurgitation >3+ at 10 years was 90 ± 3 %.

**Conclusions:**

Minimally invasive mitral valve surgery is a safe and reproducible approach associated with low mortality and morbidity, high rate of mitral valve repair and excellent late results.

## Background

Mitral valve surgery using conventional full sternotomy (FS) is the conventional approach for the treatment of the mitral valve disease. Despite this procedure has shown excellent postoperative outcomes, in the last two decades minimally invasive mitral valve surgery (MIMVS) has gained consensus among surgeons as it has provided greater patients satisfaction, maintaining the same quality and safety of the standard mitral valve surgery approach [1, 2].

According to a statement from the American Heart Association, the term “minimally invasive” refers to a small chest wall incision that does not include a FS [3]. The most common MIMVS approach is the right thoracotomy (RT) followed by the partial sternotomy. Compared with conventional surgery, MIMVS has been shown excellent results in terms of mortality, morbidities and pain, providing shorter hospital stay, faster recovery and return to normal activities which translate into less use of rehabilitation resources and healthcare costs [4–10]. Although these benefits, criticisms have been raised as MIMVS is technically more complex, requires a distinct learning curve and is associated with higher incidence of neurological events, aortic dissection, groin complications and higher rate of mitral valve replacement instead of mitral valve repair [5, 11]. Since 2003, we started our MIMVS program and after few years, RT approach has become the standard approach for the treatment of mitral valve disease. The aim of our study is to report early and long-term outcomes of consecutive patients who had undergone mitral valve surgery using RT during a 10-year period.

## Methods

### Patient selection and data collection

**Fig. 1:**
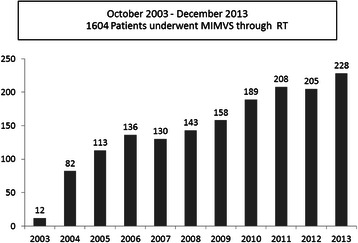
Number of minimally invasive mitral valve procedures in 10-year activity

A retrospective, observational study was undertaken of prospectively collected data in 1800 consecutive patients undergoing mitral valve surgery, of which 1604 underwent MIMVS through RT between September 2003 and December 2013 (Fig. 1). One hundred and ninety-six procedures were performed in sternotomy. The main reason for performing a sternotomy approach was the selection patient for the learning curve, and in case of very poor left ventricular ejection fraction, strong pleural adhesions, severe chronic obstructive pulmonary disease and active endocarditis with abscess involving the mitro-aortic continuity. The ethical committee approved the study, and individual consent was waived. The data collection form was entered in a local database and included three sections completed consecutively by the cardiac surgeons, anaesthetists and perfusionists involved in the care of the patients.

The main outcomes investigated were early and late mortality, perioperative complications, and freedom from MR recurrence and reoperation.

Early mortality was defined as any death occurring within 30 days of operation or before discharge from the hospital. Stroke was defined as any new focal or global neurological deficit lasting more than 24 h, and were confirmed whenever possible by neurologists and neuroradiologists. Postoperative stroke was diagnosed if evidence was found of a new neurologic deficit with morphologic substrate confirmed by computed tomography or nuclear magnetic resonance imaging.

Acute kidney injury was defined as postoperative creatinine increase of 1+ mg/dl or requirement of haemodialysis. Perioperative myocardial infarction was defined as maximum creatine kinase MB elevation three times above the upper normal level and new Q waves >0.04 ms and/or reduction in R waves >25 % in at least two contiguous leads in the electrocardiogram or the proof of new wall motion disturbances in the echocardiogram. Pulmonary complications included ventilation failure, reintubation and tracheostomy.

All patients were seen 8 to 12 weeks postoperatively and thereafter were contacted for follow-up data. The median follow-up time was 32 months (interquartile range 9–59 months) and the follow-up data were 96.6 % complete.

All patients had a preoperative transthoracic echocardiography as well as at discharge. The severity of mitral regurgitation was graded following the European Society of Cardiology and the European Association for Cardio-Thoracic Surgery recommendations [12]. Echocardiographic follow-up consisted of those patients who survived and had postoperative echocardiogram more than 6 months (812/1492, 52 %) at a median of 15 months (IQR 6–51) after surgery. Recurrent MR was defined as moderate or severe on a 4-point grade: trivial, 1 of 4; mild, 2 of 4; moderate, 3 of 4; and severe, 4 of 4.

#### Surgical technique

The standardized surgical approach for MIMVS has been reported elsewhere [13]. Briefly, MIMVS by a way of right anterior thoracotomy was performed through a 5–7 cm skin incision placed at 3th or 4th intercostal space. After incision a soft tissue retractor is inserted and the intercostal space is gently spread with a retractor. Two trocars are inserted in the thorax to allow positioning of a ventricular vent, CO2 insufflator, camera device and pericardial stay sutures. Whereas at the beginning of our experience the approach involved retrograde arterial perfusion and balloon endoclamping, the procedure has evolved to a technique with ascending aorta cannulation, long femoral venous cannula drainage, and direct transthoracic aortic clamping. Specifically, direct aortic cannulation was performed using Easyflow (Sorin, Salluggia, Italy) or Straightshot (Edwards Lifesciences, Irvine, Calif) cannulas. Biomedicus single stage (Medtronic, Minneapolis, Minn) or RAP single 2 stage cannulas (Estech) were inserted through the femoral vein into the right atrium and the correct position was achieved with the Seldinger technique under transesophageal echocardiographic guidance. In case of mitral and tricuspid valve surgery, a single 2 stage cannula (RAP, Estech) was used as it allows to drain simultaneously the superior and inferior venae cavae. After vacuum-assisted cardiopulmonary bypass ( −40 to −60 mmHg) was established, the patients were cooled to 34 C°. the ascending aorta was clamped with the Cygnet crossclamp (ovare surgical System, Cupertino, Calif) or with the aortic Glauber clamp (Cardiomedical GmbH, Langenhagen, Germany; distributed by sorin, Salluggia, Italy) and antegrade cold crystalloid or warm blood cardioplegia is delivered directly into the ascending aorta by a needle vent catheter.

The mitral valve is approached with a traditional left paraseptal atriotomy and exposed using a specially designed atrial retractor held by a mechanical harm inserted through a right parasternal port. Mitral valve procedures were performed under a combination of direct vision and thoracoscopic assistance. The surgical steps that we were unable to perform under direct vision were performed with video assistance, such as placement of sutures on the anterior annulus of the mitral valve, or at the level of the posterior medial commissures. In the setting of degenerative mitral valve disease, mitral valve repair was the first approach for the degenerative mitral valve repair; mitral valve replacement was commonly used in presence of severe annulus and/or leaflet calcifications and patients older than 80 with complex complex repair. All patients received an accurate intraoperative transoesophageal echocardiogram before and after weaning from cardiopulmonary bypass machine. In patients who had an attempt to repair, our policy is to replace the mitral valve if a) at the hydrostatic saline test after several attempts, there is still some degrees of mitral regurgitation, b) the surface of coaptation is not enough to guarantee a long durability, c) at intraoperative echo, there is more than mild mitral regurgitation.

Eight surgeons contributed to this series, with 2 of them (MG, MS) performing 59.2 % of the operations.

#### Statistical analysis

Continuous data were expressed as mean ± standard deviation or median with the interquartile range and categorical data as percentages. Cumulative survival was evaluated with the Kaplan–Meier method. All reported P values are two-sided, and *P* values of <0.05 were considered to indicate statistical significance. All statistical analyses were performed with SPSS 22.0 (SPSS, Inc., Chicago, IL, USA).

## Results

Baseline characteristics are listed in Table 1. The mean age was 63 ± 13 years, 770 (48 %) patients were female and 215 (13.4 %) had previous cardiac operations. The mean body mass index was 25 ± 4.8 Kg/m^2^. The mostpredominant pathology was degenerative disease (*n* = 1114, 70 %), followed by functional mitral valve regurgitation (*n* = 191, 12 %), rheumatic disease (*n* = 151, 9.4 %), endocarditis (*n* = 80, 5 %) and prosthetic dysfunction (3.2 %), Table 2. Mitral valve repair was performed in 1137 (71 %) patients and 476 (29 %) had mitral valve replacement (Table 2). Direct aortic cannulation was achieved in 1325 (83 %) patients. In 1432 (89.9 %) of cases, the aorta was cross-clamped; in 130 (8.1 %) an endo-aortic balloon was used, and in 43 (2.7 %), operations were performed in beating heart or ventricular fibrillation.

**Table 1:** Baseline characteristics

Variable	Overall	Repair	Replacement
	*N* = 1604	*N* = 1137	*N* = 467
Age, years (mean ± SD)	63 ± 13	61 ± 13	67 ± 11
Female sex, N (%)	770 (47.0)	493 (43.4)	277 (59.3)
Hypertension, N (%)	940 (57.3)	639 (56.2)	301 (58.6)
Diabetes, N (%)	167 (10.2)	97 (8.5)	70 (15.0)
COPD, N (%)	155 (9.5)	96 (8.4)	59 (12.6)
Previous valve operations, N (%)	156 (9.7)	37 (3.3)	119 (25.5)
Previous CABG, N (%)	59 (3.7)	30 (2.6)	29 (6.2)
Preop AF, N (%)	545 (33.9)	312 (27.4)	233 (49.9)
NYHA III-IV, N (%)	538 (33.5)	314 (27.6)	224 (48.0)
Preop EF %(mean ± SD)	57.6 ± 9.3	58.8 ± 8.6	54.7 ± 10.2
Pulmomary Hypertension (≥60 mmHg)	152 (9.5)	84 (7.4)	68 (14.5)

**Table 2:** Mitral valve pathology

Mitral valve pathology	Overall	Repair	Replacement
	*N* = 1604	*N* = 1137	*N* = 467
Degenerative	1114 (69.5 %)	907 (81.5 %)	207 (18.5 %)
Functional	191 (11.9 %)	136 (71.2 %)	55 (28.8 %)
Reumathic	151 (9.4 %)	17 (11.2 %)	134 (88.8 %)
Endocarditis	80 (5.0 %)	46 (57.5 %)	34 (42.5 %)
Prosthesis dysfunction	38 (2.4 %)	10 (26.3 %)	28 (73.7 %)
Miscellaneous	30 (1.9 %)	21 (70 %)	9 (30 %)

Median CBP time was 131 (IQR 109–162) min, and median cross-clamp time was 88 min (IQR 69–112). Concomitant procedures included tricuspid valve repair (*n* = 234, 14.6 %), atrial fibrillation ablation (*n* = 152, 9.5 %) and atrial septal defect closure (*n* = 51, 3.2 %).

### Early outcomes

Overall in-hospital mortality was 1.1 % (*n* = 19, predicted median EuroSCORE 6 %, interquartile range 3–14 %). Thirty-four patients (2.1 %) had conversion to sternotomy either because of bleeding (21, 1.3 %), strong adhesions (9, 0.5 %), aortic dissection due to the use of endoclamp (4, 0.25 %); 23 (1.4 %) patients required an IABP. Incidence of stroke, transient ischemic attack and acute renal failure requiring dialysis was 2 % (*n* = 32), 0.25 % (*n* = 4) and 1.3 % (*n* = 21). Twenty-three patients (1.4 %) had myocardial infarction, 41 patients (2.5 %) had pulmonary complications and rate of pacemaker implantation was 3.3 % (*n* = 53). Mean ICU stay was 1 day (IQR 1.0–1.0), and mean ward stay was 6 days. 951 (59.3 %) patients were discharged home. Postoperative outcomes are reported in Table 3.

**Table 3:** Early outcomes

Variable	Overall	Repair	Replacement
	(*N* = 1604)	*N* = 1137	*N* = 467
In hospital Mortality	19 (1.2 %)	4 (0.4 %)	15 (3.2 %)
Stroke	32 (2 %)	20 (1.8 %)	12 (2.6 %)
RF requiring dialysis	21 (1.3 %)	11 (1 %)	10 (2.1 %)
New onset postoperative AF	253 (15.8 %)	171 (15.0 %)	82 (17.6 %)
Reoperation for bleeding	78 (4.9 %)	48 (4.2 %)	30 (6.4 %)
Ventilation time, h	7 (5–11)	7 (5–10)	9 (6–15)
ICU stay, day	1 (1–1)	1 (1–1)	1 (1–1)
Ward stay, day	5 (4–7)	5 (4–6)	5 (4–7)
Discharged Home	951 (59.3 %)	769 (67.6 %)	182 (39.0 %)

#### Mitral valve repair

Out of 1210 candidates for valve repair, 1137 were effectively repaired (94 %), while 73 (6 %) required replacement after a repair attempt. Repair techniques included annuloplasty (95 %), either alone (264/1137, 23.2 %) or with another repair technique (814/1137, 71.6 %), including leaflet resection (63 %), neochordae implantation (16 %), and sliding plasty (11 %). For the 1137 patients that were effectively repaired, 19 (1.7 %) required conversion to full sternotomy and 29 (2.5 %) had to be reoperated for evidence, at pre-discharge echocardiography, of significant mitral regurgitation: in 12 cases the valve was replaced; in the remaining 17 (59 %) the valve was effectively re-repaired.

After repair, residual MR at discharge was very low (no MR: 803 (72,6 %), trivial: 264 (23 %), mild: 38 (3.3 %), moderate MR: 1 (0.08 %).

Out of 1114 patients affected by a degenerative mitral valve disease, 958 (86 %) of them were candidates for repair; successful repair rate was 94.7 % (907/958). Out of the remaining 156 patients, 80 were not deemed amenable to repair because of previous operation on the valve (*n* = 32) or severe calcifications (involving annulus and/or leaflets *n* = 48). In the remaining 76 cases, the learning curve was considered the main cause.

#### Mitral valve replacement

Mitral valve replacement was performed in 467 patients. The predominant pathologies were degenerative (*n* = 207, 44.3 %) and rheumatic (134, 28.7 %). Other entities were functional regurgitation (*n* = 36), ischemic regurgitation (*n* = 19), active (*n* = 24) and inactive (*n* = 10) endocarditis, prosthesis dysfunction (*n* = 28), congenital valve disease (*n* = 2), and other (*n* = 7). Mechanical and biological prosthesis was implanted in 224 (48 %) and 243 (52 %), respectively. As previously stated, 73 replacements were failed repairs.

### Late outcomes

Median follow-up time was 32 months (IQR 9–59) and was 96.6 % complete. At follow-up, 114 patients were dead: 61 patients were MVR and 53 were mitral valve repair.

#### Survival

**Fig 2:**
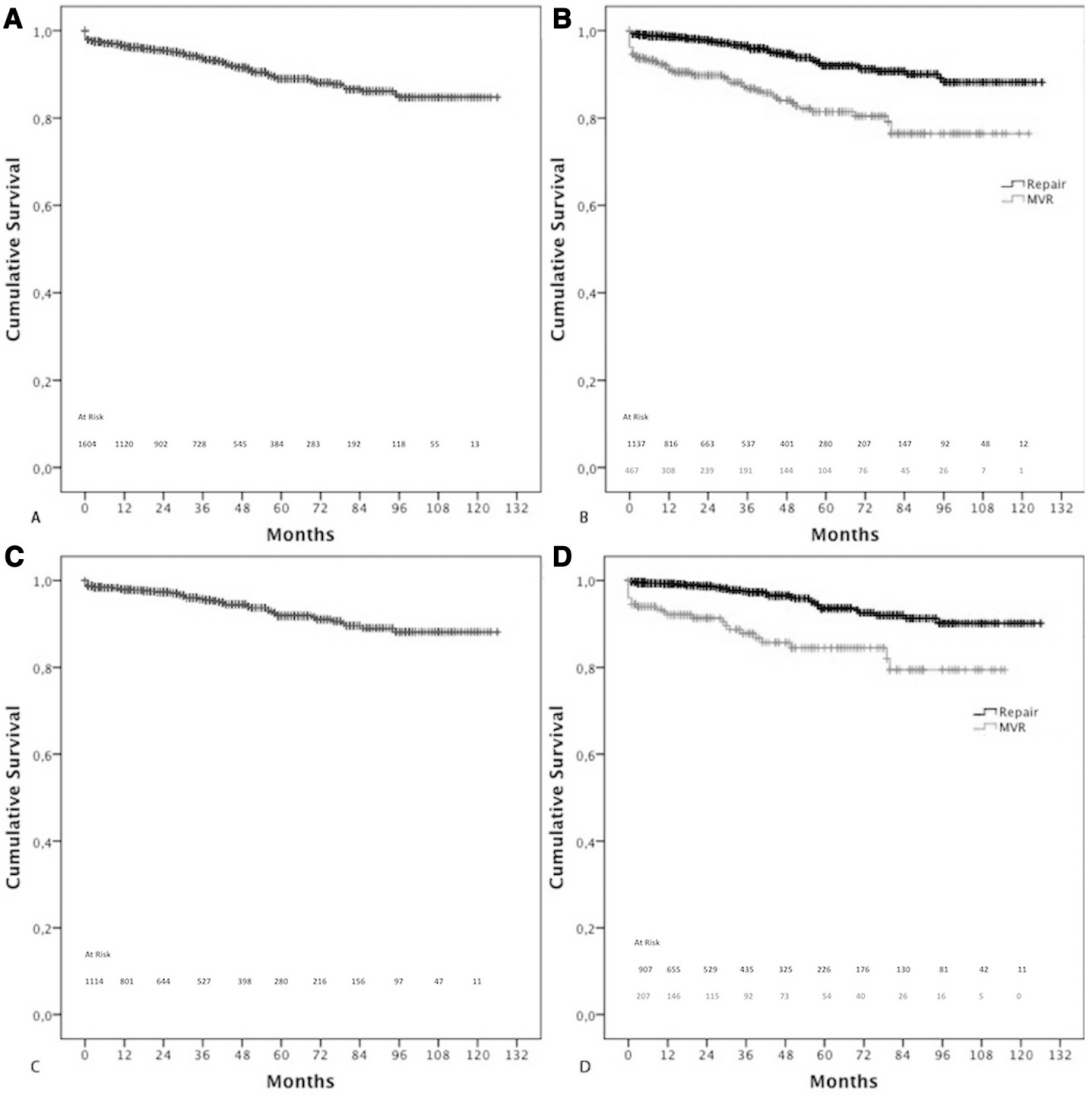
**a**: Overall survival; **b**: overall survival in mitral valve repair and replacement; **c**: overall survival in patients undergoing mitral valve surgery for degenerative mitral valve disease; **d**: survival in patients undergoing mitral valve repair and replacement for degenerative mitral valve disease

Overall 1-, 5- and 10-year survival were 96.3 ± 0.5 %, 88.9 ± 1.1 %, and 84.5 ± 1.8 % respectively, Fig. 2a. Specifically, survival after repair at 1-, 5- and 10-years was 98.5 ± 0.4 %, 91.9 ± 1.2 %, and 88.0 ± 2.1 % respectively. Survival after replacement at 1-, 5- and 10-years was 91. ±1.4 %, 81.3 ± 2.5 %, and 76.2 ± 3.4 % respectively, Fig. 2b.

In the setting of degenerative mitral valve diseased, overall 1-, 5-, and 10-year survival was 98.1 ± 0.4 %, 91.8 ± 1.2 %, and 88.1 ± 1.9 % respectively, Fig. 2c. Specifically, rate of survival after mitral valve repair was 99.2 ± 0.3 % at 1 year, 93.5 ± 1.3 % at 5 years and 90.1 ± 2 % at 10 years, whereas survival after mitral valve replacement was 92.7 ± 1.8 % at 1 year, 84.4 ± 3.2 % at 5 year, and 79.3 ± 4.6 % at 10 year, respectively Fig. 2d. Overall 1-, 5- and 10-year survival were 94.3 ± 1.7 %, 83.8 ± 3.8 % and 81.2 ± 4.5 % for patients who received a mechanical valve and 88.3 ± 2.2 %, 83.8 ± 3.8 % and 81.2 ± 4.5 % for those who had a bioprosthesis.

#### Reoperation

**Fig 3:**
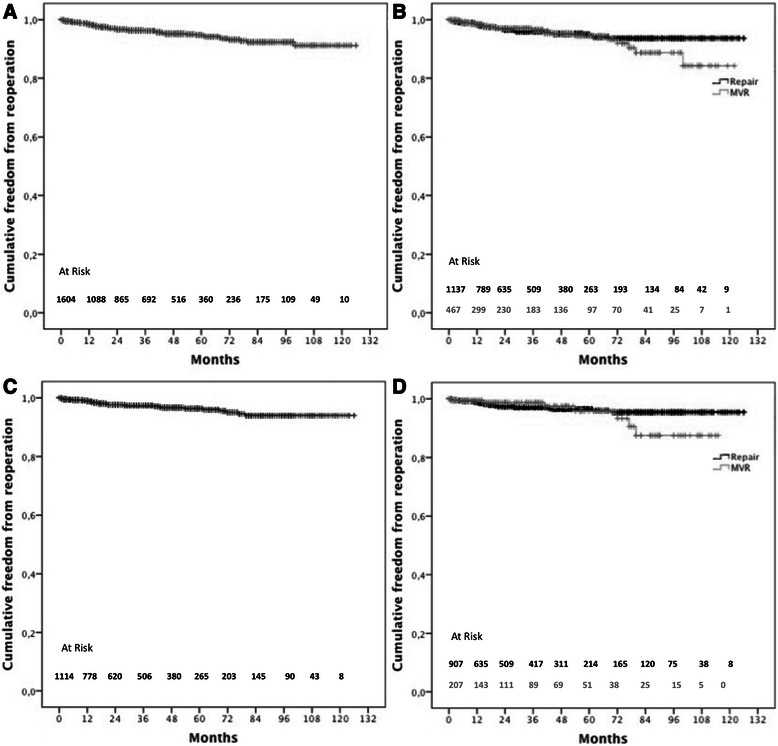
**a**: Overall freedom from reoperation; **b**: overall freedom from reoperation in mitral valve repair and replacement; **c**: overall freedom from reoperation in patients undergoing mitral valve surgery for degenerative mitral valve disease; **d**: overall freedom from reoperation in patients undergoing mitral valve repair and replacement for degenerative mitral valve disease

Overall freedom from reoperation was 98.6 ± 0.3 %, 94.7 ± 0.7 %, and 91.1 ± 1.7 % at 1-, 5-, and 10-years, respectively, Fig. 3a. After mitral valve repair, freedom from reoperation at 1-, 5- and 10-years was 98.4 ± 0.4 %, 94.8 ± 0.9 % and 93.6 ± 1.1 %, respectively. Freedom from reoperation after replacement at 1-, 5- and 10-years was 98.6 ± 0.6 %, 94.5 ± 1.6 % and 83.9 ± 5.5 % respectively, Fig. 3b.

In the setting of degenerative mitral valve disease, overall 1-, 5-, and 10-year freedom from repair was 99 ± 0.3 %, 96.3 ± 0.8 %, and 93.9 ± 1.3 % respectively, Fig. 3c. Specifically, rate of freedom from reoperation after mitral valve repair was 98.9 ± 0.3 % at 1 year, 96.4 ± 0.8 % at 5 years and 95.4. ± 1.1 % at 10 years, whereas aftermitral valve replacement was 99.5 ± 0.5 % at 1 year, 95.8 ± 2.3 % at 5 year, and 91.3 ± 5.1 % at 10 year, respectively Fig. 3d.

#### Recurrent mitral regurgitation

**Fig. 4:**
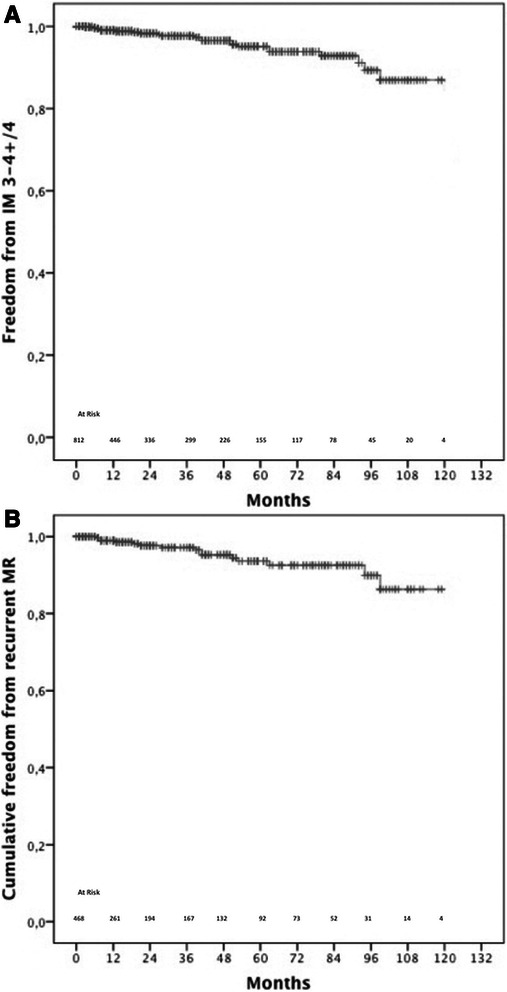
**a**: Overall freedom from recurrent MR; **b**: freedom form recurrent MR in patients undergoing undergoing mitral valve repair for degenerative mitral valve disease

Freedom from recurrent mitral regurgitation was 99 ± 0.4 % at 1 year, 95 % ± 1.3 % at 5 years and 86.6 % ± 3.8, Fig. 4a. In the setting of patients undergoing mitral valve repair for degenerative mitral valve disease, freedom from recurrent MR after 1, 5 and 10- years was 99 ± 0.6 % and 93.6 % ± 1.9 %, 86 % ± 4.9 %, Fig. 4b.

## Discussion

We demonstrated that MIMVS through RT is a safe procedure, associated with excellent postoperative outcomes, short hospital length of stay and outstanding long-term results. Specifically, overall in-hospital mortality was 1.1 % lower than the recent mortality rate reported in the Society of Thoracic Surgeons Database and the low incidence of postoperative complications as well as the high long-term survival, highlight the safety of the this procedure in all settings of mitral valve disease [1]. Finally, in the setting of all mitral valve repair success rate at discharge was 93 %, with a freedom from reoperation of 94 % at 10 years. Similar results were found for the patients undergoing mitral valve repair for degenerative mitral valve disease.

MIMVS has been shown to decrease postoperative complications, providing fast recovery, shorter hospital length of stay, less pain, better aesthetic appearance and consequently less use of hospital resources [8]. In 2010, the consensus statement of international society of minimally invasive cardiothoracic surgery (ISMICS) concluded that MIMAVS may be an alternative to conventional mitral valve surgery, given that there was comparable short term and long term mortality, comparable in-hospital morbidity (renal, pulmonary, cardiac complications, pain perception and readmissions), reduced sternal complications, transfusions, postoperative AF, duration of ventilation and ICU and hospital length of stay [5]. Similar results were described by the Society of Thoracic Surgeons of the adult cardiac surgery database as well as by several meta-analyses confirming the main points of the aforementioned consensus statement [4, 6–8, 14]. Our results are in line with the current literature; however, despite these excellent outcomes, many criticism still remain regarding MIMVS as it is technically more complex, requires a distinct learning curve (prolonged cross-clamp and cardiopulmonary bypass times) and is supposedly associated with higher incidence of neurological events, aortic dissection, groin complications and higher rate of mitral valve replacement instead of mitral valve repair [5, 11]. Some authors argue that limited exposure may lead to insufficient de-airing and thus an increase risk in neurologic events [14]. We believe that our standard technique, with central cannulation and direct aortic cross-clamping is as safe as sternotomy in terms of neurological events. We report a 2 % incidence of postoperative stroke and similar results have been reached by others [10, 15–17]. It has been shown that the incidence of stroke does not seem to depend on the surgical access [8, 16], but rather on the choice of endo-clamp and peripheral vessel cannulation [4, 17, 18]. Previously, we demonstrated that the use of retrograde perfusion is associated 4-fold increase in stroke and postoperative delirium when compared to anterograde perfusion. We strongly believe in antegrade perfusion as it is more physiological, reduces neurological events and avoid the morbidities related the femoral arterial cannulation in terms of pseudoaneurism and wound dehiscence. In addition, the use of flexible cannulae in ascending aorta e direct cross clamp has abolished the problem of aortic dissection, described in previous studies [4–6]. In our series, two patients (0.1 %) experienced aortic dissection with the use of endoaortic ballon occlusion. It has been suggested that limited exposure may compromise probability of mitral valve repair when performing MIMVS. Overall, data from STS database and from the Euro Heart Survey indicate that the rate of valve repair in the US and Europe was 41 and 46.5 % respectively [19, 20]. Recently, Gammie reports a 67 % repair rate through median sternotomy and 85 % repair rate through minimally invasive access [14]. Many series have been published so far documenting excellent MIMVS repair rates (overall 81.2 %, on intention-to-repair basis 98.4 %), even in the setting of Barlow’s disease (94.5 and 100 %) [9, 21, 22]. We report 94 % repair rate of valves that were deemed amenable to repair and 94.7 % in the setting of degenerative valve disease; we are more keen to attribute repair failures to the complexity of the underlying disease rather than to insufficient exposure. In our series, low residual MR at discharge and outstanding freedom from reoperation indicate excellent standards of repair, in line with other studies, once again confirming that access through minithoracotomy does not interfere with quality and durability of valve repair [8, 9]. Finally, regarding the learning curve, a cross sectional survey on MIMVS concluded that at least more than 20 cases are required to gain familiarity with this procedure and at least one case needs to be performed per week to maintain proficiency [23]. Since 2005, in our institution, MIMVS has become the standard approach for the treatment of mitral valve disease. Therefore, due to the low numbers of standard sternotomy mitral procedures we have been forced to introduce young surgeons to mitral valve surgery directly through a minimally invasive approach. Recently a CUSUM analysis performed on seven surgeons has shown that MIMVS can be safely performed with low morbidity and mortality, even in mitral valve repair [24, 25].

The main limitation of this study is its retrospective nature; however our database is prospectively compiled. Although we reported excellent long-term results, echocardiographic data were not available for all patients. No information has been recorded regarding the aetiology of perioperative myocardial infarction as well as no information has been reported regarding the cause of late mortality. Finally we did not have the possibility to compare our series with a control group, as since the 2005 MIMVS is our standard approach, and patients demand less invasive procedures. Nevertheless a well-designed study with appropriate sample size is required to validate the advantages of MIMVS.

## Conclusions

Minimally invasive mitral valve surgery is a safe and reproducible approach associated with low mortality and morbidity, high rate of mitral valve repair and excellent late results.

## Abbreviations

MIMVS: minimally invasive mitral valve surgery
RT: right anterior minithoracotomy
